# Flatboats, Travelers, Infectious Diseases, and Other River Thoughts

**DOI:** 10.3201/eid2201.AC2201

**Published:** 2016-01

**Authors:** Byron Breedlove

**Affiliations:** Centers for Disease Control and Prevention, Atlanta, Georgia, USA

**Keywords:** art science connection, emerging infectious diseases, art and medicine, about the cover, Thinking River Thoughts: Flatboats, Travelers, Infectious Diseases, The Jolly Flatboatmen, George Caleb Bingham, luminism, sexually transmitted infections, public health

**Figure:**
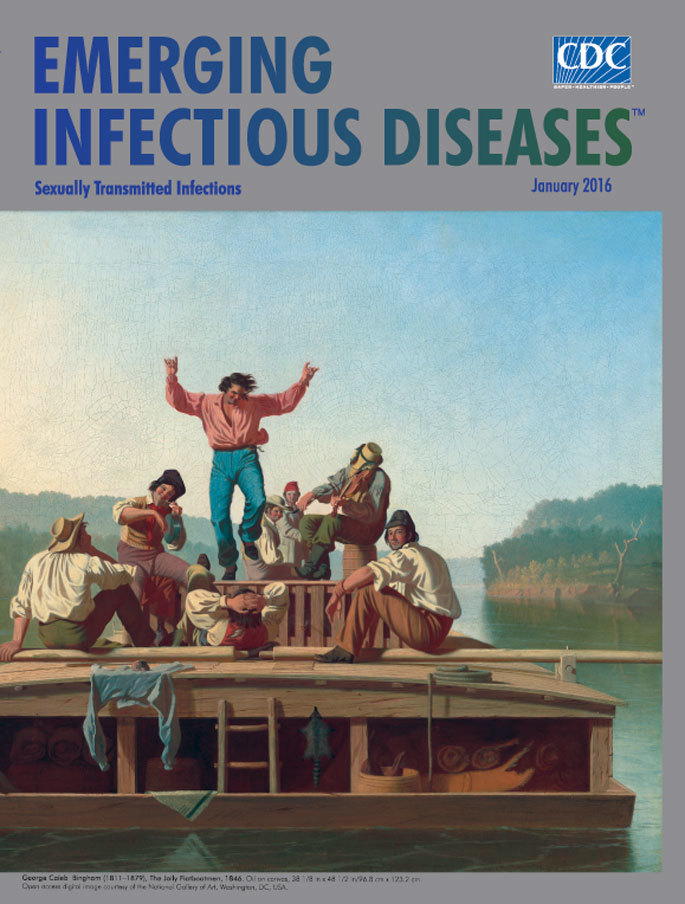
**George Caleb Bingham (1811–1879), *The Jolly Flatboatmen*, 1846. Oil on canvas, 38 1/8 in x 48 1/2 in/96.8 cm x 123.2 cm.** Open access digital image courtesy of the National Gallery of Art, Washington, DC, USA. Patrons’ Permanent Fund. 2015.18.1.

“I choose to listen to the river for a while, thinking river thoughts, before joining the night and the stars.”

—Edward Abbey, *Down the River*

A constellation of synonyms exist for the word “river”: what distinguishes these naturally flowing watercourses from a beck, bourn, brook, burn, creek, rill, rivulet, runnel, or tributary may come down to the size of the stream or may simply be a regional preference. Rivers flow across every continent and on all but the smallest islands, carving, eroding, and reshaping the Earth’s topography; connecting populations; enabling access to natural resources, commerce, and trade; defining boundaries; and offering sustenance and energy. Rivers have provided routes for explorers and adventurers and have roused the imaginations of writers and artists across cultures and history.

An iconic depiction of life on North American rivers in the 1840s, *The Jolly Flatboatmen*, this month’s cover image, is one of the most celebrated works by American painter George Caleb Bingham. This well-preserved painting is considered an early example of luminism, an American painting style that depicts the effects of light played out across tranquil settings, calm waters, and hazy skies. Drifting downriver, Bingham’s happy-go-lucky crew pays little mind to their surroundings: nothing in this painting hints at the potential dangers of river life—shifting sandbars and shoals, flash flooding, submerged trees, injuries and diseases, or scheming pirates.

The National Gallery of Art’s overview states that “The composition is at once dynamic—the dancing man and the musicians—and elegantly stable in the way Bingham arranged the figures to form an isosceles triangle.” The Gallery, which has borrowed and displayed this painting several times since the 1960s, finally purchased it from an undisclosed seller in May 2015.

The painting’s symmetry is striking, a point noted in a review of Bingham’s river paintings from the *New York Times*: the rectangular flatboat floats downstream, framed by a peaceful river, misty tree-lined riverbanks, and a pale blue, cloudless sky. A pair of long oars, locked in place, jut off to either side. Front and center, a capering young man, dressed in a red shirt, dances a jig, frozen in mid-step with hands held high. One crew member plays a fiddle, and another keeps time on a pan. Other crewmen watch and listen, several locking eyes with the viewer; another, seen from behind, stretches out in repose, his head resting on his interlocked hands.

A raccoon pelt hangs by the ladder; a serpentine coil of rope dangles from the top deck; a turkey thrusts its head between the slats of the wooden crate doubling as a stage; a rock anchors the blue shirt drying; bed rolls are tidily stashed below the top deck. Draped over the back of the boat, a sheet of newspaper and its reflection in the river form additional, smaller triangles. According the National Gallery of Art, Bingham’s meticulous attention to details helped establish the artist’s enduring reputation.

Bingham, who was born in Virginia and raised in Missouri, began his career as a self-taught portrait painter. In the mid-1840s, he started painting his genre works that idealized labor and leisure on the river. Even though flatboats had become essentially obsolete by that time, Bingham nonetheless saw his reputation soar when *The Jolly Flatboatmen* became the first piece of American art that, to borrow from today’s social media lingo, “went viral.” In 1847, the American Art-Union purchased the painting from the artist and distributed engravings to some 10,000 members across the United States, which made it one of the best known and most widely distributed works of art of its era.^1^

For a time, vast numbers of flatboats, forerunners of today's barges, conveyed agricultural commodities, raw materials, whiskey, livestock, and people downriver. Stops along the way served to increase contact between local populations and the flatboat crews and travelers. Those contacts and human behaviors created opportunities for rapid and efficient transmission of many types of pathogens—including those that can cause sexually transmitted infections. Infectious diseases could be transmitted from geographically isolated populations to more densely populated communities and, conversely, from urban populations to susceptible, isolated populations

Today the number of people who travel for work, leisure, and adventure has exponentially increased, and global mobility contributes to the spread of sexually transmitted infections. The *CDC Health Information for International Travel* (the Yellow Book) notes that an estimated 499 million cases of chlamydia, gonorrhea, syphilis, and trichomoniasis occur worldwide each year. Emerging and reemerging sexual infections, including antimicrobial resistant gonorrhea, chancroid, sexually transmitted hepatitis C, lymphogranuloma venereum, HIV infection, and human papillomavirus infection, further underscore the value of heightened public health surveillance and research.

## Footnotes

1 From 1839 through 1851, the American Art-Union promoted American art by distributing engravings of the best American paintings of the day—including works by George Caleb Bingham, Thomas Cole, Jasper Francis Cropsey, Asher B. Durand, and William Sidney Mount—to its nationwide membership.
